# American Medical Association

**Published:** 1878-07

**Authors:** 


### EDITORIAL.

### Recent Meeting of the American Medical Association.

The great American Medical Association has passed over Buffalo, "and the gates are shut." Never in the history of the Association has there been a more harmonious, useful and pleasant meeting. The scientific and practical were freely commingled with the social and legislative, so that the varied talent of the members was afforded ample opportunity, and we believe all enjoyed an unmixed pleasure. Every one seemed glad to look his neighbor in the face, and many pleasant acquaintances and desirable friendships were formed. The social opportunities were fully appreciated and enjoyed, and we think every physician present was made wiser and better.

We notice some of the Councilors of the Massachusetts Medical Society think it hardly worth while for that Society to provide for appointment of delegates annually to the American Medical Association, because, as one of them said, "one can't tell how long that body will continue in existence."

We think we can understand how this impression was formed. The recent meeting was favored by the presence of some of the most worthy and distinguished physicians of that State, who, in attending receptions given by the Buffalo Club, Prof. and Mrs. James P. White, Fine Arts Association and Society of Natural Sciences, finally reached the residence of Bronson C. Rumsey, Esq., on Delaware Avenue, and after polite and cordial reception by Mr. and

Mrs. Rumsey, passed immediately to the magnificent grounds, when, with but slight aid of the imagination they at once concluded that the American Medical Association had been suddenly and most unexpectedly admitted bodily into the Kingdom of Heaven, and of course reported that it would not "continue longer in earthly existence." But seriously, it was no great stretch of fancy for Mr. Rumsey's grounds, lighted with many colored lanterns, shining through the trees and down upon the lake, to be taken for enchanted land; angels looking down from model temples upon nymphs floating in magnificent gondolas upon the smooth waters of the lake below; fountains forming cascades over rocky beds; music floating down from the balconies above; hundreds of gentlemen and ladies in princely costumes filling the winding avenues of the park, and every recess in the elegant mansion, and making the less imaginative turn to their companions and ask, "What is this; who and where are we?" "Is it possible that this was made by human hands, or is this the true city of the new Jerusalem, with its gates of pearl and its streets of gold, and no need of the light of the sun?" All these questions were answered by another sip of "nectar," and the existence of the American Medical Association was settled for a long time to come.

But to return, the election of officers was an item of interest to many, and announcement being made of time for election of state representatives, every delegate hurried into some corner or chair, stairway or alcove and cried, delegates from New York, delegates from Pennsylvania, delegates from Illinois, &c., &c., with the wind of an Eastern Cyclone. One of the number would elect himself to mount a chair, stool or bench, and begin to make a speech about how President should be elected, which no one could hear. Each crowd was naming nominating member for the State, amidst confusion and consternation. In about three minutes some one previously agreed upon was declared nominating committee by some one or two screaming wildly aye, aye, aye, and the President of the Am. Med. Association was made. With all this disorder and indiscrimination the lot fell upon a very worthy and distinguished representative of the Am. Med. Profession, and we all shouted the louder aye, aye, aye.

This has worked all very well for the Association thus far. Representative men by merit of being from the North, South, East or West, have filled the place of coveted honor (?). It has been proposed to change the manner of electing officers. We have but a word on this subject. If there are any men the American Medical Profession desire to honor we hope for change in this respect, as at present the *honor*, as it seems to be regarded, is a very questionable one, and should not be left thus to so great indiscrimination and uncertainty.

———:o:———

We are indebted to the daily press of Buffalo for the data of our report of American Medical Association proceedings, and the entire Medical press of the country will join with us in expression of thanks.

Lactopeptine.—This preparation which has the merit of being considerably cheaper than the best kinds of Pepsin, has been found by actual experiment to possess a decided and uniform solvent power, greater, weight by weight, than Pepsin as usually prescribed. It is a combination of Pepsin, Sugar of Milk, Pancreatine, Ptyalin, and Lactic and Hydrochloric Acids. We have administered Lactopeptine in a number of cases where Pepsin was indicated, and have been fully satisfied with the result.—*New York Medical Journal.*

